# Do virtual patients prepare medical students for the real world? Development and application of a framework to compare a virtual patient collection with population data

**DOI:** 10.1186/s12909-017-1013-1

**Published:** 2017-09-22

**Authors:** M. Urresti-Gundlach, D. Tolks, C. Kiessling, M. Wagner-Menghin, A. Härtl, I. Hege

**Affiliations:** 1 Institute for Medical Education, University Hospital of LMU Munich, Munich, Germany; 2 Brandenburg Medical School Theodor Fontane, Neuruppin, Germany; 3 Medical University Vienna, Teaching Center, Vienna, Austria

**Keywords:** Virtual patients, Healthcare system, Authenticity, Medical education

## Abstract

**Background:**

An important aspect of virtual patients (VPs), which are interactive computer-based patient scenarios, is authenticity. This includes design aspects, but also how a VP collection represents a patient population and how a patient is presented in a VP scenario. Therefore, our aim was to analyze VP scenarios integrated into the combined internal medicine and surgery curriculum at the University of Munich (LMU) and compare the results with data from the population in Germany.

**Method:**

We developed a coding framework with four main categories: patient data, patient representation, diagnoses, and setting. Based on the framework we analyzed 66 VP and compared the results with data from the German healthcare system.

**Results:**

Especially in the categories of patient data and patient representation, the VPs presented an unrealistic image of the real world; topics such as unemployment, disability, or migration background were almost non-existent. The diagnoses of the VPs and the onset of diseases were comparable with the healthcare data.

**Conclusions:**

An explanation for the lack of representativeness of the patient data and representation might be a trend to create VPs based on fictional patient stories with VP authors trying to minimize complexity and cognitive load for the students.

We suggest raising awareness among VP authors concerning personalized representations of patients without overwhelming their students. Our framework can support educators to assess the authenticity and diversity of a VP collection.

## Background

Virtual patients (VPs) can be defined as "specific type of computer-based program that simulates real-life clinical scenarios; learners emulate the roles of health care providers to obtain a history, conduct a physical exam, and make diagnostic and therapeutic decisions" [1]. VPs have been integrated into undergraduate healthcare curricula for many years as self-directed learning activities [2] to teach clinical reasoning skills [3] in blended-learning scenarios [4] or for assessment purposes [5]. VPs show a great variety concerning design aspects such as interactivity, feedback, instruction, or provision of information [6]. Additionally, authenticity is a major aspect [7, 8], because VPs are typically designed to provide a “real-life clinical scenario”, that is adapted to the learners level of competence and curricular needs. A meta-analysis identified three main factors influencing the learner’s perceived authenticity: authenticity of the VP story, the format of the VP, and the quality of the computer representation [9]. In focus groups medical students emphasized the authenticity of the web interface, of the learner tasks, and the use of media to represent the patient as important aspects of VP design [10]. For the design of VPs authentic and fictive material can be combined, for example by enriching an invented patient story with media or data from real patients [11].

VPs, especially when provided for self-directed learning, are typically part of a larger collection of VPs. According to Shaffer and Resnick the concept of a “thick” authenticity includes "learning that is authentic in its relation to the real world outside the school" [12]. Thus, in addition to authenticity-related aspects of each VP, we should consider the VP collection as a whole and its relation to the world outside medical school; this encompasses how well a VP collection represents a relevant patient population - for example in terms of diversity, age and gender distribution, diagnoses, setting of the clinical encounter, or how the virtual patients are represented as persons.

Both, the context of a clinical scenario and probabilities of diseases play an important role in clinical reasoning [13, 14] and we believe that these aspects should be considered when shifting the teaching from the real world to the virtual world, unless there is a didactical reason for deliberately designing a non-representative VP collection. For example, if in a self-contained VP collection for the training of clinical reasoning skills rare diseases are unintendedly overrepresented this could leave a wrong impression about the probability of such a rare disease and impact student’s development of heuristics.

Comparable to other educational scenarios VP collections may deliver a “hidden curriculum” and convey unintended messages to the learners [15]. For example, Turbes et al. showed that in a collection of case examples the overall pattern of demographics and lack of information were inconsistent with their formal multicultural curriculum [16].

At the medical school of Ludwig-Maximilians-Universität (LMU), similar to other medical schools, a collection of VPs based on curricular objectives has been integrated for self-directed learning into the curriculum across various disciplines [17, 18].

Our aims were to develop a coding framework to analyze VP collections and subsequently apply it to the VPs, that are embedded in the integrated medicine and surgery year at the LMU medical school in terms of two aspects:

1. How well does the VP collection represent the German population?
2. How is the patient represented in the VP scenarios?

## Methods

### Developing the coding framework

We developed the coding framework based on a purposive literature review, statistical data from the German healthcare system, and the experience of the authors with case-based learning, virtual patients, and healthcare systems. In discussion rounds among our interdisciplinary group of two medical educators, a clinician, a psychologist, a public health expert, and a medical student the codes were defined and described. We divided the coding schema (Table 1) into the four main categories patient data, patient representation, diagnoses, and setting.

**Table 1:** Overview of the coding frame. The full coding schema, including examples and more detailed descriptions can be obtained upon request

Codes	Description	Values
Patient data		
Sex		Male/female/other/not available (NA)
Age	Age of the patient at the time of the first encounter	In years/NA
Sexual orientation		Heterosexual/ Homosexual/Other/ NA
Body-Mass		Body-Mass-Index (BMI)/NA
Disability	Any physical or mental disability	Yes/No/NA
Occupation	Occupation of the patient at the time of the first encounter	Free text/NA
Cultural, language, or migration aspect	Any cultural aspects, language barriers or migration aspects that could influence the patient encounter	Yes/No/NA
Substance abuse (smoking, alcohol, drugs)	Any substance (ab-) use, such as smoking, alcohol, or illegal drugs	Yes/No/NA
Patient representation (adapted from Kenny et al. [32])		
Name	The patient is introduced with a name and is addressed by name	First name/last name/NA
Social context	Family, relatives, or friends of the patient are mentioned	Family, friends/everyday life
Use of direct speech and dialogs	Representation of the communication with the patient in dialogs and direct speech	Free text/NA
Expression of concerns and emotions	The patient expresses concerns or emotions	Free text/NA
Use of multimedia	Representation of the patient with multimedia elements	Image/video/text/NA
Diagnoses		
Number of diagnoses	Number of diagnoses the patient is/was suffering from	Yes/No/NA
Diagnosis/−es	Main/final diagnoses	22 ICD-10 (International Statistical Classification of Diseases and Related Health Problems) chapters 2015 [36]
Onset	Acute or chronic onset of symptoms	Acute (<= 1 month), chronic (>1 month) [37]
Setting		
Setting (initially and during the course of the scenario)	Setting of the first encounter with the patient and transferal to other settings	University hospital, hospital (non-university), general medical practice, specialist, non-medical facility, other, NA (single choice)
Scenario closure	Concluding of the VP scenario	Ongoing, resolved, died, unstated [37]

To count and code the diagnoses of a VP the final diagnosis/−es and any relevant secondary or past diagnoses (e.g. if mentioned in the past medical history) were extracted from the VP description. The diagnoses were coded as specific International Statistical Classification of Diseases and Related Health Problems (ICD-10) diagnoses, summarized as ICD-10 chapters (e.g. “Neoplasm” or “Diseases of the respiratory system”), and finally sorted based on the frequency.

### Setting and usage of virtual patients

Medical students at LMU Munich learn internal medicine and operative medicine in an integrated curriculum during their second and third clinical term. The courses include face-to-face teaching such as lectures, problem-based learning sessions, and bedside-teaching. Additionally, a collection of 66 online VPs is provided as a self-directed learning module. The VPs are designed to match curricular objectives of common symptoms, train clinical reasoning skills, and complement the face-to-face courses. The VPs were created with the VP system CASUS® [19] by different authors and have been reviewed by a content matter expert and a medical educator. A faculty development course about didactical aspects of VP creation is offered at LMU and didactical guidelines are available within the VP system. However, authors were not specifically instructed to construct the VPs based on real patients or focus on common vs. rare diseases.

### Coding

After obtaining consent from the course instructors responsible for the 66 VPs, we exported and downloaded the VPs from the CASUS VP system as PDF files. One author (MU) coded all VPs based on the coding guide, which in this process was further elaborated and enriched with examples and more detailed descriptions. Finally, three authors (DT, CK, IH) each coded 33% of the VPs. Inconsistencies were discussed among the authors and resolved by consensus; if necessary the coding guideline was adapted accordingly.

The data were collected into a Microsoft Office Excel 2011 spreadsheet and further analyzed using SPSS (version 22).

## Results

### Patient data

Thirty-three VPs (50.8%) were female, 32 (49.2%) male, in one VP this information was missing. According to the statistical Federal Office in 2015, 49.1% of the German population were male and 50.9% female [20].

**Fig. 1:**
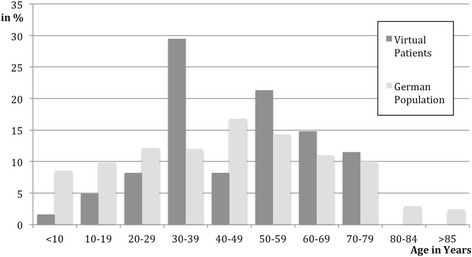
Age distribution of VPs (*n* = 61) and the German population in 2010 [38]

The age distribution of the VPs compared to the German population is shown in Fig. 1, in 5 VPs this information was missing.

None of the VPs included a non-heterosexual orientation of the patient. In surveys coducted in 2008 about 83% of participants in Germany described themselves as heterosexual [21].

**Fig. 2:**
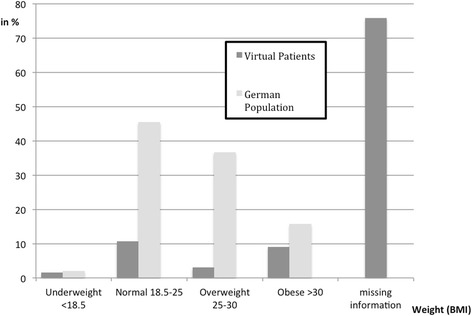
Overview of the average Body-Mass-Index (BMI) of the VPs and the German population (sample census 2013) [39]

In 16 VPs (24.2%) the Body-Mass-Index (BMI) of the patient was included in the scenario description or could be calculated based on weight and height (Fig. 2); in all other cases it was not mentioned at all or only vaguely described (e.g. “a slim patient”).

One VP (1.5%) was described as intellectual disabled; in all other cases no physical or mental disability was mentioned. In 2013 the percentage of disabled persons in the German population was 12.7% [22].

In 40 VPs (60.6%) the description of the patient included his/her occupation. From these 12 (30.0%) had an academic occupation, such as physician or engineer and 12 (30.0%) were pensioners or housewives (no house husbands). None of the VPs were described as unemployed, compared to a 6.7% unemployment rate in Germany in 2014 [23].

One VP (1.5%) covered a migration aspect with a guest worker from Bosnia ("The communication with the patient turns out to be difficult, because he does not speak any German."); other migration aspects, such as refugees or asylum seekers were not covered. According to the Federal Statistical Office in 2014 the German population included 20.3% persons with a migration background [24].

In 15 VP scenarios (22.7%) a smoking history of the patient was included; 80% of these were smokers (actual smoker or smoking history) and 20% non-smokers. According to the Federal Statistical Office the percentage of smokers in the German population was 24.5% in 2015 [25]. The existence or absence of a substance abuse (alcohol or other drugs) was mentioned in six VPs (9.1%). Two of these VPs included a pathological consumption of alcohol, in three VPs the patients negated the consumption of alcohol and in one VP the consumption of drugs was negated. A survey of substance abuse in 2015 showed that about 2% of the German population were addicted to alcohol and about 1% were addicted to other drugs [25].

### Patient representation

Thirty-four VPs (51.5%) were addressed with their full name, 25 (37.9%) with their last name, and seven (10.6%) did not have a name. 36 VPs (54.5%) included an image of the patient and in nine VPs (13.6%) the patient was represented in a video or a series of videos. 27 VPs (40.9%) did not provide any media material of the patients’ appearance.

A social context, either family or everyday life context, was described in 41 VPs (62.1%). In 44 VPs (66.71%) dialog format or direct speech was used to represent the patient’s communication; in 34 VPs (51.5%) the patient talked about his symptoms in direct speech. In 39 VPs (59.1%) the patient expressed emotions and concerns.

### Diagnoses

Forty-two VPs (63.6%) covered one or two diagnoses; on an average 56.4% of the German population suffers from one or two diagnoses [26]. We were unable to determine the number of diagnoses in two VPs because information was missing. In one VP (1.5%) the patient was discharged as healthy after a needle stick injury. Main reasons for the hospitalization of patients were diseases of the circulatory system, followed by injuries and poisoning (Table 2).

**Table 2:** Overview of the distribution of the five most frequent final diagnoses (based on ICD-10 2015 chapters) in the VPs (*n* = 77 final diagnoses) and the German healthcare system based on the Statistical Federal Office [28]

	Most frequent VP diagnoses	Most frequent diagnoses in the German healthcare system
1	Diseases of the circulatory system	Diseases of the circulatory system
2	Injury, poisoning and certain other consequences of external causes	Injury, poisoning and certain other consequences of external causes
3	Neoplasms Diseases of the respiratory system	Diseases of the digestive system
4		Neoplasms
5	Diseases of the musculoskeletal system and connective tissue	Diseases of the musculoskeletal system and connective tissue

Thirty-six (54.5%) of the VPs described an acute onset of a disease, whereas in 22 (33.3%) VPs the patient suffered from a chronic disease; in eight VPs (12.1%) the patient suffered from a complication of an underlying chronic disease. According to the Robert-Koch Institute, 40.8% of patients in Germany suffer from a chronic disease in 2012 [27].

### Setting

The setting of the VP scenario was described in all 66 VPs. In 41 VPs (62.1%) the scenario started in a hospital setting, including university hospitals; 14 VPs (21.2%) initially took place in a medical practice, and eleven VPs (16.7%) outside of a medical facility, such as the patients’ home. 50 VPs (75.8%) were resolved at the end of the scenario, 16 (24.2%) were ongoing; none of the VPs died. The death rate of hospitalized patients in Germany was 2.1% in 2014 [28].

## Discussion

We developed a coding framework to analyze the VP collection that is provided to medical students in the interdisciplinary year at LMU Munich and evaluate (1) its authenticity compared to the German population and (2) how the patient is represented as a person. The results revealed three interesting aspects - missing information, realistic image of the German population, and patient representation, which we will discuss in more detail.

### Missing information

Many VP authors did not provide basic information about the patients’ social history, such as occupation, sexual orientation, smoking history, alcohol or drug consumption, or risk factors such as the patients’ BMI. In some VPs this information would have been relevant for the clinical decision-making. Presumably, authors attempted to reduce the amount of information to adapt the complexity of the VP to the students’ level of expertise. However, authentic and sufficient information allow the learner to better identify with the scenario [29]. VP authors could benefit from more guidance and standardization in the VP creation process, either provided by the VPs system or by authoring guidelines or VP creation courses addressing such issues and how to balance authenticity vs. cognitive load.

### Image of the German population

The VPs represented the reality closely in terms of frequency of diagnose categories, number of diagnoses, and onset of symptoms. This indicates that the creation process based on curricular objectives was successful. Also, the gender distribution was comparable to the population in Germany.

However, in other aspects the VP collection was not a representative sample of the German population; patients with a disability or a migration background and unemployed or dying patients were almost non-existent in the VP collection.

This lack of representativeness may be caused by the authors’ effort to simplify the VP scenario, either by overemphasizing relevant factors or by omitting challenging aspects. Alternative explanations could be that authors were unaware of the importance of including diversity or felt uncomfortable describing more challenging situations.

The age distribution showed an unrealistic peak of VPs aged 30–39. Interestingly, this age peak coincides with the age of the VP authors, who were mainly residents in that age range. Thus, authors might have a natural tendency to develop and describe virtual patients who are similar to themselves.

Further research is needed to investigate these questions.

Overall, the authenticity of the VP collection could be enhanced by including aspects, such as diversity, managing challenging situations as explicit learning objectives for the VPs and a didactic concept should encompass both, the VP and VP collection. Authors should be aware of their own personal situation, biases, or viewpoints when creating VPs, especially when VPs are constructed based on fictive patient stories [30].

Additionally, authors and curriculum managers should be aware of the “hidden curriculum” and the possibility of conveying unintended messages by creating unrepresentative VP collections as described by Turbes et al. [16].

### Patient representation

The patient is an essential actor in a VP scenario, and we strongly believe this should also be reflected in an appropriate representation. More than 10% of the patients in the VP scenarios were not addressed with a name and more than 40% were not represented with an image or video. A focus group study by Huwendiek et al. showed that students prefer an image of the patient at the beginning of a VP scenario to a textual representation; they associate multimedia elements with higher authenticity [10], and a study by Kamnin et al. found that students learning from cases with videos showed enhanced critical thinking compared to text [31].

Kenny et al. emphasize the importance of personalizing and appropriately valuing patients in text-based scenarios by using active voice and a social context [32]. In the VP collection over 60% of the VPs provided dialogs to represent the patient’s communication and included a social context; in almost 60% of the VP scenarios the patient’s emotions or concerns were included. Overall, we suspect a trend to depersonalize patients in VP scenarios; reasons might be unawareness or efforts and obstacles to personalize a VP, such as obtaining a patient’s consent to take a picture or record an interview.

Various guidelines for VP creation have been published and provide valuable tips for authors (e.g. Posel et al. [33] or Begg [34]). However, in our opinion they do not sufficiently address the real-world authenticity of a VP collection. We suggest expanding such guidelines to include tips about implementing a realistic VP collection in terms of patient and context diversity.

### Limitations

Our study has several limitations. First, it has to be noted that the comparisons of the VP collection with the data from the German healthcare system are based on different populations and years. The VPs represent an adult patient population, whereas some of the data from the German healthcare system are based on the overall population. However, we believe that the comparison is meaningful and the discrepancies would be even higher when comparing the VP data with the patient population in Germany.

Secondly, we focused on the interdisciplinary year (internal medicine and surgery) and did not analyze all VPs that are included in the curriculum of the medical school at LMU. Although unlikely, expanding the analysis to VPs of all content domains may change the results.

Thirdly, we were unable to determine retrospectively how many VPs were based on a real or fictive patient story and how many authors attended the VP authoring course or consulted the guidelines. Therefore, future research is needed to investigate the influence of a real vs. fictive patient story and of different VP creation trainings on the authenticity and diversity of a VP collection.

## Conclusions

VP systems have lowered the technical burden for healthcare educators to create virtual patients by providing sophisticated user interfaces and guidance. However, there is a need for raising awareness among VP authors of a personalized representation of the patient and how their own experiences and environment may influence the VP scenario. In addition, curriculum managers should be aware of the real-world authenticity of a VP collection provided to students compared to the healthcare system; especially aspects such as disabilities, migration background or unemployment should be considered.

We believe that our coding framework to assess the authenticity and diversity of a VP collection provides a valuable aid for educators to analyze and improve their VP collections. Further research is needed to investigate the influence of the VP creation process on the authenticity of a VP collection. Analyzing larger VP collections, such as the international eViP repository [35] of more than 350 VPs, could provide more insight.

## Abbreviations

BMI: Body-Mass-Index
eViP: Electronic Virtual Patient Project
ICD, ICD-10: International Statistical Classification of Diseases and Related Health Problems
LMU: Ludwig Maximilians University Munich
NA: Not available
VP: Virtual Patient
